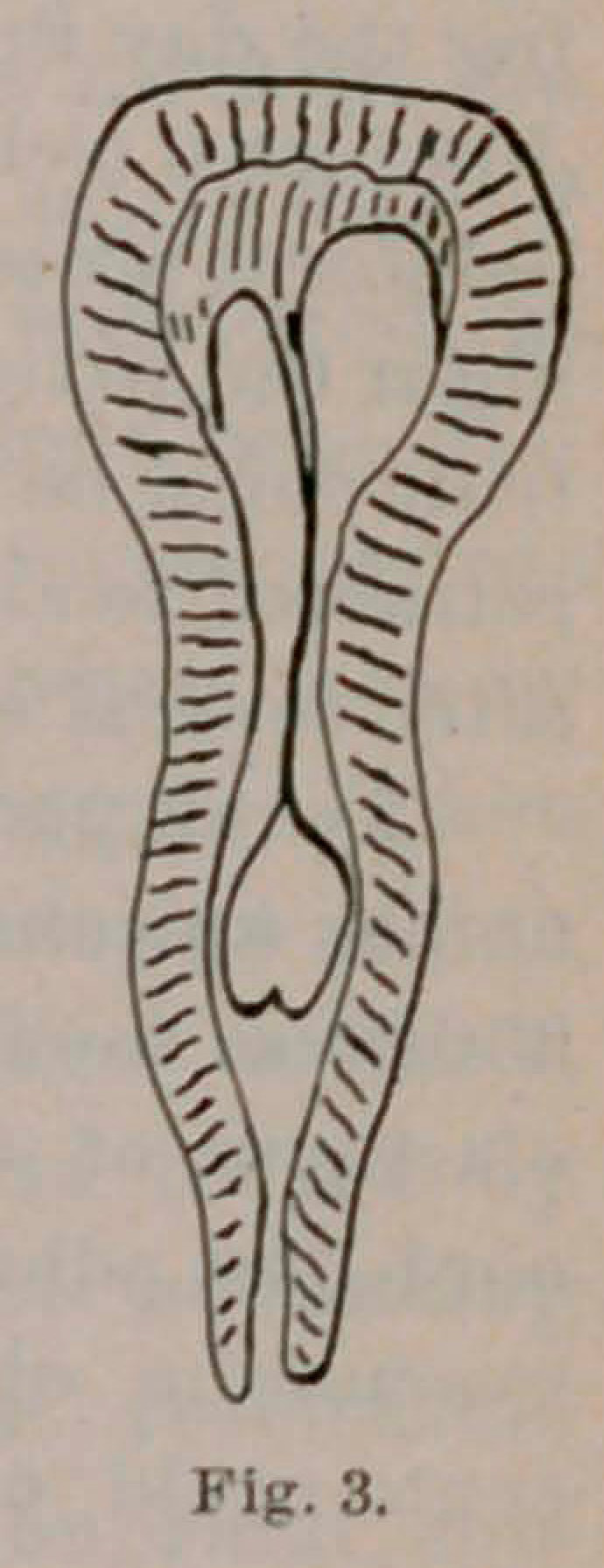# A Case of Retroflexion of the Gravid Uterus

**Published:** 1896-06

**Authors:** Gabriele Baronin Possanner

**Affiliations:** Vienna. Wasagasse 2


### BUFFALO MEDICAL JOURNAL.

VOL. XXXV. JUNE, 1896. No. 11.

### Original Communications.

### A CASE OF RETROFLEXION OF THE GRAVID UTERUS

### COMPLICATED BY HYPERTROPHY OF THE PORTIO SUPRAVAGINALIS AND PERIMETRITIC ADHESIONS.

From the I. Obstetric University Clinic of the Professor, Dr. F. Schauta, in Vienna.

BY DR. GABRIELE BARONIN POSSANNER, Vienna.

Translated by DR. MAUD E. ABBOTT, Montreal.

IN THE study of obstetrics I have often remarked the relatively slight attention given by most authors to the influence of anomalies of the soft passages upon the course of labor. This fact is still more surprising when one considers the frequency of the occurrence of such anomalies on the one hand and on the other the importance of this influence on the course of labor. Led by these considerations, I have accepted the invitation of my honored chef professor, Dr. Schauta, to describe a case of this kind which I had the opportunity of closely observing in his wards. I have been still more encouraged to do this by the fact not only that this case is in itself an extremely interesting one but also because I have not been able to find any report of an exactly similar case in the whole range of accessible literature :

Frau T. K., aged 21, III. para, of Vienna, was admitted to the clinic on May 1, 1894, with the above diagnosis. The personal history gives the following facts: Until three years ago the patient had been always healthy; neither has she, as far as can be ascertained, a hereditary predisposition. Menstruation began at the age of twelve years, was always regular every four weeks ; it was accompanied by colicky pains in the abdomen and lasted from three to four days. Three years ago the patient had suffered an abortion, which was followed by an attack of puerperal childbed fever, lasting six weeks. Frau K. was at that time under treatment in the Rudolf hospital. Cold douches were used and cold applications to the abdomen, whereupon the patient's condition improved greatly, so that, according to the statement, she was fit to be dismissed after six weeks' treatment. In January, 1893, took place, at the normal end of pregnancy, the spontaneous birth of a fully-developed living child; but again, during the lying-in period, an inflammation of the uterus set in, which obliged the patient to seek admission at the Wilhelminen hospital. Again douches and cold applications to the abdomen were ordered, and the patient states that after six weeks' treatment she was discharged "cured," and felt perfectly well until three months ago. At that time pains set in, in the lower part of the abdomen, on both sides. The patient also complains since then of a feeling of pressing down and burning in the abdomen, and menstruation has not occurred since that time. The pains ceased during rest in bed in the dorsal position and increased greatly with every active movement, especially upon exertion of the abdominal muscles, as in defecation and also in the passage of urine, which does not take place without a certain amount of straining. About three weeks ago a slight hemorrhage took place from the genitals, directly after the patient had lifted a heavy weight and at the same time she noticed a small tumor protruding from the vulva. Since that time the patient has had a feeling as though the womb had fallen.

*Status clinicus* on May 1, 1894.—Medium-sized, well-nourished woman. The thoracic organs show normal condition. The mucous membrane of the genital organs is discolored, livid and there is a slight discharge. Before the vulva lies a tumor (prolapse) the size of an apple, of a bluish-red color; on the portio are places covered with a dirty deposit. The corpus uteri is distinctly enlarged and in retroversion. The column is very much elongated. The corpus uteri corresponds in size to a pregnancy of the fourth lunar month. Internal examination shows the portio elongated and deeply lacerated. A very soft tumor, about the size of a child's head, is felt in Douglas's sac. The fundus uteri is not palpable; both adnexa are normal; the corpus uteri can easily be raised out of Douglas's sac.

On May 7th.—The uterus extends four finger breadths above the symphysis and has the consistence of a pregnant uterus; there is tenderness to pressure over the tumor and on its lateral borders. The tumor protruding from the vulva shows a transverse fissure; its anterior surface is of a scarlet color, granular and passes over posteriorly into a smooth surface.

With regard to the etiology it may be remarked that the puerperal illnesses described by the patient are to be considered as attacks of perimetritis, as remains of which adhesions exist, through which the uterus is fixed. The portio vaginalis, respectively supravaginalis, which is, in consequence of the blood and lymph congestion, extremely elongated and hypertrophied, cannot advance upward, because of the fixation, and must therefore, in consequence of the pressure of the continually enlarging pregnant uterus, give way below as the point of least resistance. (Fig. 2.)

The retroflexed uterus can now be replaced only with the greatest difficulty and the pessary used after replacement falls out again after twenty hours. Quite as unsuccessful was the application of wadding tampons, supporting bandages, and the like, the execution of which the patient went through with the greatest patience.

In the course of the patient's stay in the clinic the prolapse became considerably larger, in spite of continuous rest in bed on her part, and at the same time her sufferings increased greatly. Especially in the last week of May, tormenting drawing pains set in, in the hypogastric region, which however ceased from time to time. The ulcer on the

Fig. 1.

Fig. 2.

Fig. 3.

portio, which had been covered before, cleaned soon under treatment with a solution of acetate of alumen.

On June 1st.—Strong bearing-down pains in the lower part of the abdomen set in. On this account, and also because at the same time marked difficulties in micturition occurred and the portio threatened to become necrotic, the inducement of artificial abortion was resorted to. At examination on June 1st, at 12 A. M., the following condition was found : the uterine tumor stood 1 fingerbreadth below the umbilicus ; respectively, 4 fingerbreadths above the symphysis—that is, exactly as high as at the first examination. The prolapse protruded from the vulva. Its anterior wall measures 10.5 cm. ; its lateral walls, each 7 cm. ; its posterior wall, 8 cm. The whole portio is covered with a single, circular, deeply-granulated erosion, measuring 7 cm. in sagittal diameter, 6 cm. in transverse diameter. The circumference of the prolapsed cervix is 18 cm., the color of the mucous membrane was light pink, that of the erosion dark brownish red. On internal examination, firm, tense cords, running backward from the upper part of the cervix, were felt, which were very sensitive to pressure. In the same way such cords were felt in the left parametrium ; the right parametrium was in its whole extension much shortened and very resistant.

Inducement of artificial abortion was undertaken on June 1st, at 12.30 P. M., by the introduction of an elastic bougie. The bougie entered easily and without resistance as far as to its end. An iodoform dressing was then applied. Toward evening slight labor pains set in. On June 2d, removal of the first and introduction of a fresh bougie. Toward evening, pains again set in. The night, however, was free from pain. In the morning, strong, persistent pains and some passing of blood. Bougie removed. During the night of the 3d of June there were moderate labor pains. In the morning of June 4th the prolapsed part appeared markedly shortened and somewhat bluish, discolored. During the day there had been very little abdominal pain. On the 4th of June, at 6 o'clock in the evening, introduction of a new bougie and an iodoform protecting bandage was applied. Throughout the following night the patient had very strong pains. On June 5th, 12.30 P. M., the fruit could be felt, about 3 cm. from the external os. The prolapse had retracted to about 3.5 cm. (Fig. 3.) On the 5th of June, at 2.30 P. M., after several strong labor pains the fruit was at last born, head first.

As the placenta was not yet delivered at 8 o'clock in the evening, and as a profuse hemorrhage began, manual extraction was resorted to. This was found to be very difficult on account of the length and slight elasticity of the cervix (9 cm.) (Fig. 1), and of the extremely firm, cord-like adhesions of the placenta to the fundus uteri. Finally entire evacuation of the uterus was successfully carried out, but the placenta could only be removed by pieces. When the removal of the placenta was finished, another hemorrhage set in, which was stopped by an irrigation with lysol and tamponade of the uterine cavity.

On June 6th the prolapse had entirely withdrawn itself through the vulva, also remained replaced during the following days. In order to assist involution of the hypertrophy by moderate pressure, as well as to induce fixation in the normal position, the vagina was well tamponed, iodoform gauze and a protective bandage applied. The tamponade was renewed twice a day. There was very little secretion.

On June 10th the tampon was pushed out by the prolapse before the vulva. The tampon was renewed. General condition was very good. A stool followed an enema without any difficulty.

On June 11th the patient complained of painfulness of the cervix during the introduction of the tampon. The tamponade was therefore stopped. In spite of this the prolapse still continued replaced during complete rest in the dorsal position.

During the following days pains of every description had completely disappeared, the patient felt very well and the prolapse remained, without tamponade, continually in position. Lochia very scanty, purulent.

Examination on July 4th gives the following conditions: portio hypertrophic, the size of a walnut, lacerated, external os a transverse fissure, closed, not eroded. Posterior fornix of vagina very sensitive to pressure. Passing backward from the cervix cord-like resistances are plainly felt, which are also sensitive to pressure. The uterus is in retroflexion, is however easily replaceable, it is somewhat enlarged, of normal consistence, adnexa on both sides normal. During defecation or other straining of the abdominal muscles the anterior wall of the vagina and the cervix prolapse as far as the introitus vaginal, without however passing out of it. Condition of the patient, otherwise, perfectly normal.

If we now sum up the above-described case, it may be stated in a few words somewhat as follows: A 21-year-old patient had, three years ago, an abortion, fifteen months ago a birth at the normal end of pregnancy. Each time a perimetritis set in during the lying-in period, which yielded to the treatment employed, at least as far as acute symptoms are concerned. Patient was dismissed both times cured, feeling perfectly well. The consequences of both puerperal illnesses first became apparent when a new pregnancy occurred and enlargement of the uterus began. Then pains set in, in the back and abdomen, caused by the tension which the already somewhat tense and stretched adhesions suffered through the upward extending uterus. The uterus was forced by the progress of pregnancy, at the impossibility of advancing upward, to give way below, at the point of least resistance. Marked blood and lymph stagnation, caused by the abnormal conditions of pressure, developed, especially in the lower parts of the uterus, and in consequence the great hypertrophy and elongation of the portio vaginalis, respectively supravaginalis, ensued. At the same time the portio is continuously pushed downward and appears, apparently in consequence of a straining of the abdominal muscles, before the vulva. In addition to the congestion and abnormal pressure, mechanical insults to the greatly hypertrophied portio followed, an erosion of great extent takes place, which at the time of the patient's admission to the hospital was covered by diphtheritic masses. The deposit disappears very soon under treatment with aluminum acetate, but the erosion, in spite of the most careful treatment, not only persists, but also increases greatly, so an extensive part of the surface of the portio appears scarlet and very uneven. Finally, incarceration symptoms set in, the portio threatens to become necrotic, and in consequence artificial abortion was then induced. Noteworthy still is the mode of exit of the fruit, respectively, the condition of the portio during labor. The latter became, indeed, much shortened, could however not retract completely over the fruit, which must therefore widen the passage by degrees under the influence of strong labor pains. The third stage—placental period—lasts exceptionally long and must be ended by operation. In childbed, recovery rapidly advances, the portio decreases markedly, erosion soon disappears completely, there remains only a moderate hypertrophy of the portio and a slight prolapsus uteri.

WASAGASSE 2.

## Figures and Tables

**Fig. 1. f1:**
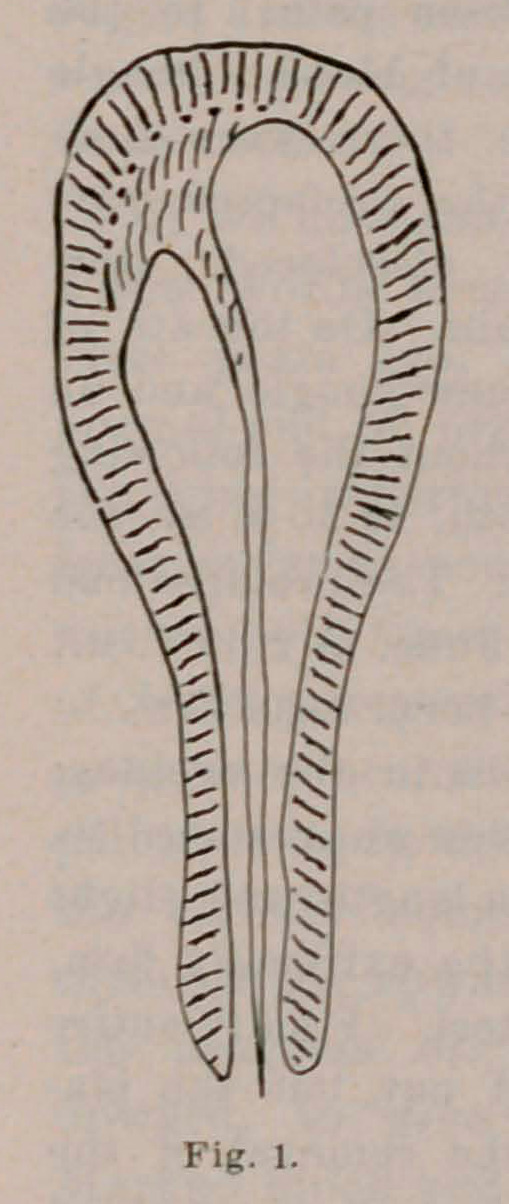


**Fig. 2. f2:**
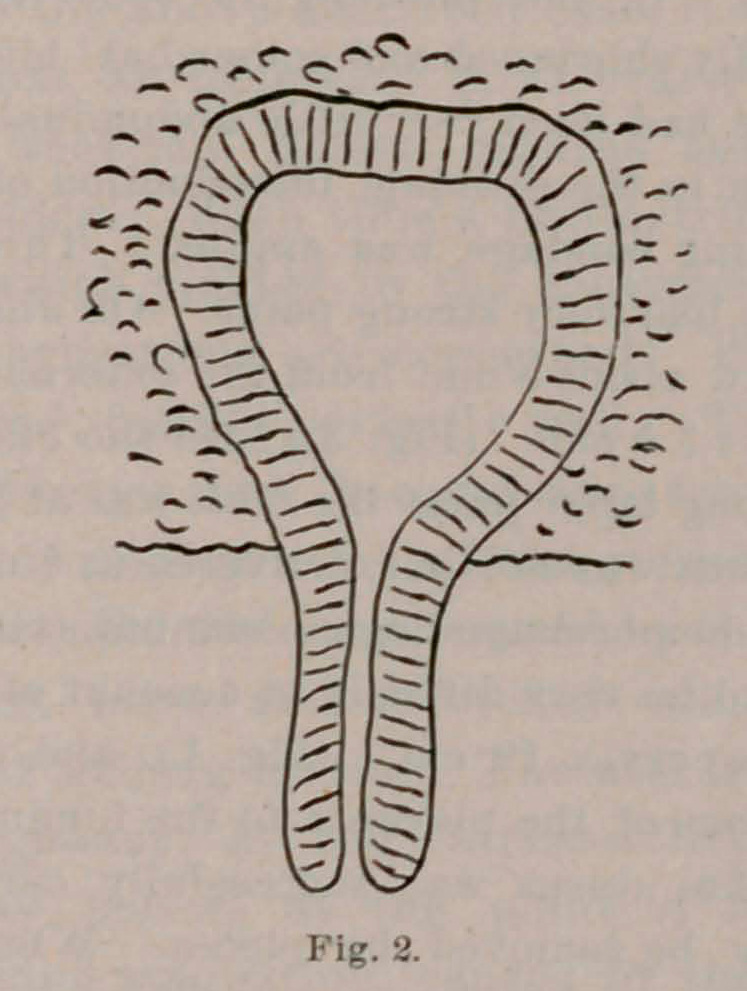


**Fig. 3. f3:**